# Cholesterol Efflux Pathways Regulate Myelopoiesis: A Potential Link to Altered Macrophage Function in Atherosclerosis

**DOI:** 10.3389/fimmu.2014.00490

**Published:** 2014-10-13

**Authors:** Andrew James Murphy, Dragana Dragoljevic, Alan Richard Tall

**Affiliations:** ^1^Haematopoiesis and Leukocyte Biology, Baker IDI, Melbourne, VIC, Australia; ^2^Department of Immunology, Monash University, Melbourne, VIC, Australia; ^3^University of New South Wales, Sydney, NSW, Australia; ^4^Division of Molecular Medicine, Department of Medicine, Columbia University Medical Center, New York, NY, USA

**Keywords:** atherosclerosis, hematopoiesis, cholesterol efflux, monocytes, macrophages, HDL

## Abstract

Atherosclerotic cardiovascular disease is a chronic inflammatory disease of the blood vessels that can lead to myocardial infarction or stroke. The major cell in the atherosclerotic lesion, the macrophage, is thought to be an important contributor to the production of inflammatory mediators that exacerbate this disease. Macrophages are generally derived from circulating monocytes, which are in turn produced by hematopoietic stem and multipotential progenitor cells (HSPCs) in the bone marrow and other medullary organs. Recent studies suggest that disruption in cholesterol homeostasis or prolonged exposure to a hypercholesterolemic environment can influence HSPCs to over-produce monocytes, resulting in monocytosis. These monocytes may carry a pre-programed ability to become M1-like macrophages once they enter the atherosclerotic lesion. Future studies may help to differentiate the role of such pre-programing versus responses to local environmental cues in determining M1, M2, or other macrophage phenotypes in atherosclerotic lesions.

Innate immunity has long been considered a driving force in the initiation and progression of atherosclerotic cardiovascular disease (CVD) (1). Indeed, inflammation is a process that has attracted considerable attention as a potential therapeutic target in atherosclerosis. It has also become well accepted that cholesterol metabolism is intimately linked to inflammation and innate immune processes. This close relationship is not only important in the effector cells of atherosclerotic disease such as monocytes (2) and macrophages (3) but cholesterol metabolism has also been shown to play a central role in their hematopoietic precursors (4, 5). This is important as changes in cholesterol homeostasis in the hematopoietic stem and multipotential progenitor cells (HSPCs) control the rate of production of monocytes/macrophages, and possibly have an influence on their function (4–6). Increased numbers of circulating monocytes are a predictor of cardiovascular risk (7–14) and studies in mice have shown a causal role (4–6, 15, 16). These key studies in mice have also revealed that HSPCs can mobilize from the bone marrow (BM) to extramedullary sites such as the spleen (4–6, 17), where they can also produce monocytes that contribute to atherogenesis (6). In this article, we will review these topics and also explore the hypothesis that the mechanisms contributing to monocyte production from HSPCs could also influence the type and function of lesional macrophages.

## Innate Immune Cell Production and Cardiovascular Risk

Monocytosis is associated with CVD and atherosclerotic plaque severity in prospective and cross-sectional human studies (7, 11–13). Monocytosis is also closely linked to plasma lipids, where a positive correlation is observed with total cholesterol levels (15, 18–20), and an inverse correlation with plasma high-density lipoprotein (HDL) levels (11, 14, 20, 21). Gerrity et al. first suggested that excessive monocyte production contributed to atherogenesis in rabbit and pig l models of hypercholesterolemia and atherosclerosis (18, 19). These studies also made the link between hypercholesterolemia and enhanced monocyte production from the BM using colony-forming assays and suggested that this could be driving the atherogenic phenotype (18). Through the use of mouse models, a causal relationship between monocyte levels and severity of atherosclerotic lesions has been shown (4, 5, 15). Studies employing the *op/op* mouse that carries a mutation in the gene encoding macrophage colony-stimulating factor (CSF-1; M-CSF) have a gene dose-dependent decrease in monocyte levels that is reflected by smaller atherosclerotic lesions (22). Conversely, western diet (WTD)-fed *Apoe^−/−^* mice display monocytosis that is proportionate to the length of feeding and reflects the size of the atherosclerotic lesion (15). We have also shown that monocytosis, largely independent of activation, accelerates atherosclerosis in mouse models (4). In addition to the abundance of monocytes that circulate, the site of production may play an important role, as monocytes produced in the spleen appear to have an atherogenic phenotype (6). While monocytes may directly contribute to atherogenesis by secreting inflammatory cytokines, ROS, and proteases, their most important role is probably to act as precursors lesional macrophages. Macrophages are a heterogeneous population of cells and have been categorized into two main groups known as M1 and M2. This classification is based on function and the expression on a number of genes. M1 macrophages are thought to be inflammatory cells, expressing a gene signature including *iNos*, *IL-6*, *Tnf-*α, and *IL-1*β, while M2 cells are thought to play a resolving role and expression genes such as IL-10, Tgf-β, and *Arg1*. However, these cells due retain plasticity and can sit at various points along the scale [see recent reviews on suggested nomenclature (23, 24)]. In *Apoe*^−/−^ mice, CCR2^+^ Ly6-C^hi^ monocytes preferentially enter the atherosclerotic lesion (15, 16), and this monocyte subset has been suggested to differentiate into a macrophage with an inflammatory phenotype. Interestingly, lesional macrophages can also undergo local proliferation to sustain their population within the advanced atherosclerotic plaque (25). The phenotype of proliferating macrophage or its product cells has not yet been studied in detail; however, as the proliferation of these cells is dependent on SR-A (25), it could perhaps be of the M2 variety (26). Below we will discuss how defects in cholesterol metabolism pathways influence the HSPCs, monocytes, and macrophages to promote atherosclerosis, and will make the speculative suggestion that events in the hematopoietic stem and progenitor populations may influence the ultimate functions of the macrophage.

## Cholesterol Efflux Pathways Link HSPC Proliferation, Monocyte Production, and Atherosclerosis

Impaired cholesterol efflux has long been associated with atherosclerosis, and more recently, the ability of HDL to promote efflux from cholesterol loaded cells was shown to be a stronger predictor or atherosclerotic burden than HDL cholesterol or apoA-I levels (27). In line with this is the experimental evidence in animal models of atherosclerosis where increasing HDL levels either therapeutically (rHDL infusions) (28) or genetically (ApoA-I transgene) (5, 29) is protective. This is thought to be due to the ability of HDL or ApoA-I to prevent foam cell formation, inhibit leukocyte adhesion, and protect the endothelium from activation (30, 31). However, recent studies have shown that HDL via cholesterol removal from the cell membrane can regulate the production of innate immune cells (4, 5, 32), particularly monocytes, by acting on HSPCs (4, 5). In respect to the anti-atherogenic properties of HDL, this may be an important function that could affect the types and/or functions of the downstream cells that eventually mature into lesional macrophages.

The removal of cholesterol from HSPCs can be facilitated by a number of pathways. We discovered that HSPCs express *Abca1*, *Abcg1*, and *Apoe* at high levels and these key efflux genes could further be induced *in vivo* by the administration of Liver-X-Receptor (LXR) agonists (4). Co-deletion of two key cholesterol efflux genes ATP bind cassette transporter (ABC) *A1* and *Abcg1*, in the hematopoietic compartment and transplantation into *Ldlr*^+/−^ mice resulted in prominent monocytosis and neutrophilia, which was accompanied by a dramatic acceleration in atherosclerotic lesion formation (5). A myeloproliferative phenotype was suggested, as myeloid cells infiltrated many major organs, including the spleen, liver, and intestine. Mice with *Abca1/g1* KO BM had a dramatic expansion of the HSPCs, which were proliferating at higher rates compared to mice that received WT BM. The enhanced proliferation in the *Abca1/g1* KO HSPCs was found to be due to an increase in the expression of the common β subunit of the IL-3/GM-CSF receptor (IL-3Rβ; aka CD131), making these cells more sensitive to these cytokines. Promoting cholesterol efflux with an apoA-I transgene reversed the proliferative defects and reduced the severity of the atherosclerosis. *Abca1^−/−^*, *Abcg1^−/−^*, and *Apoe^−/−^* HSPCs also mobilized into the circulation in increased amounts and established extramedullary hematopoiesis in the spleen and other organs (17). These sites of extramedullary hematopoiesis provide an important reservoir for monocytes in acute coronary disease (33), highlighting the multiple links between hypercholesterolemia, defective cholesterol efflux pathways, and the over-production of monocytes and neutrophils that contribute to atherosclerosis.

**Figure 1:**
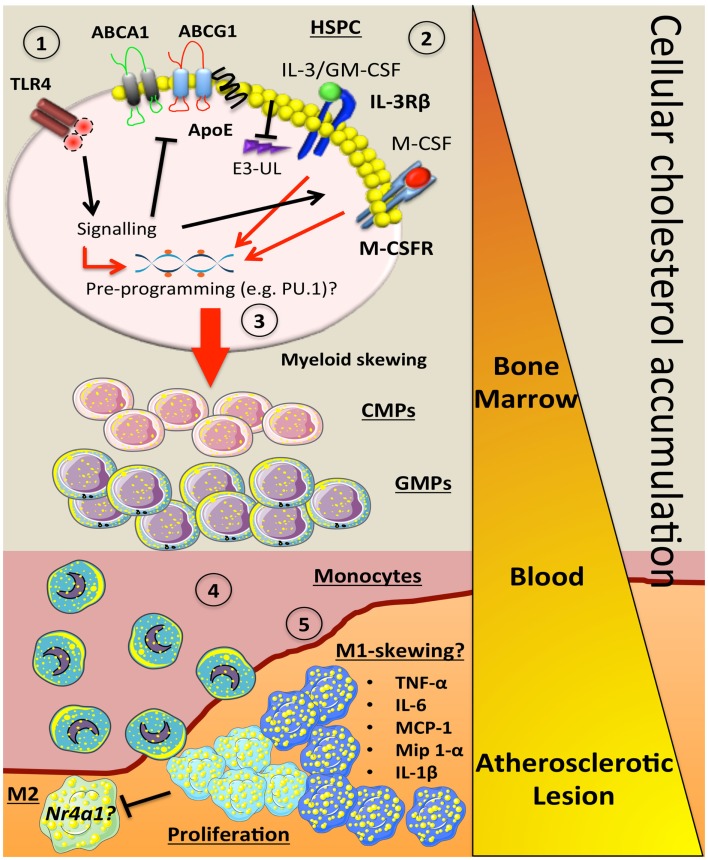
**How alterations in cholesterol metabolism and myeloid skewing contribute to atherosclerosis**. In the setting of hypercholesterolemia, inflammatory signals could be sensed by receptors such as TLR4 on HSPCs to trigger a number of downstream signaling events. This could (1) inhibit key cholesterol efflux pathways (ABCA1, ABCG1, ApoE), which would result in cellular accumulation. The increase in membrane cholesterol could lead to (2) increased cell surface expression of cytokine receptors such as IL-3Rβ and the M-CSFR due to a failure to activate E3-ubiquitin ligases (E3-UL). (3) Sustained signaling from myeloid cytokines (IL-3, GM-CSF, M-CSF) along with the hypercholesterolemic environment could pre-program the HSPC via PU.1 to produce more myeloid cells. As these cells mature in to CMPs and GMPs, they have the potential to carry more cellular cholesterol if their cholesterol efflux pathways are suppressed. (4) Once the blood monocyte is circulating, more lipid is acquired and it can carry this into the atherosclerotic plaque. (5) These lipid-laden monocytes could then differentiate into an M1-like macrophage that can also undergo local proliferation, which enhance inflammation by producing a number of cytokines and chemokines. These M-1 cells may also have a defect in *Nr4a1* and lack the ability to convert into M-2 resolving macrophages. Triangle indicates cellular cholesterol accumulation as the myeloid cells mature (orange to yellow).

As mentioned above, we and others have also reported that WTD-fed *Apoe^−/−^* mice display prominent monocytosis (4, 15, 16). We found that this was also due to expansion and proliferation of the HSPCs as a result of increased expression of the IL-3Rβ. Treating *Apoe^−/−^* mice with reconstituted HDL (rHDL; CSL-111) to promote cholesterol efflux normalized this proliferative defect (4). The role for the IL-3Rβ in promoting HSPC proliferation and monocytosis in *Apoe^−/−^* mice was confirmed in mice with deficiency of both genes (34). Through the use of competitive BM transplant (cBMT) studies, we found that these efflux pathways at least partly functioned in a cell intrinsic manner (4). For example, deletion of *Apoe* in cells marked by CD45.2 produced more monocytes and lesional macrophages compared to WT cells marked by CD45.1 that were transplanted into the same recipients. We also found that the *Ldlr^−/−^* mice that received the mix of *Apoe^−/−^*(CD45.2)/WT(CD45.1) had larger lesions compared to those that received WT(CD45.2)/WT(CD45.1). The increase in lesion size was independent of monocyte activation and supports the idea that increased production of monocytes directly impacts lesion monocyte/macrophage content, size, and severity. However, we speculate that other explanations may be involved, including that increased entry of *Apoe^−/−^* monocytes results in macrophages that have an altered phenotype/function, or that alterations in cholesterol metabolism in HSPCs pre-program their daughter cells (i.e., monocytes and macrophages) into an inflammatory phenotype (Figure 1).

To further explore the contribution of cholesterol efflux in macrophages versus HSPCs, cell specific knockouts of *Abca1/Abcg1* have been employed to examine the role of these transporters in cells down stream of HSPCs (35). Using the Lysozyme M Cre mouse crossed with *Abca1^flox/flox^ Abcg1^flox/flox^* mice (Mac-DKO), Westerterp et al. (35) were able to reduce the expression of Abca1/Abcg1 by approximately half in the granulocyte-macrophage progenitors (GMPs) and monocytes, and by ~90% in macrophages. Transplantation of the *Mac^ABCDKO^* BM into *Ldlr^−/−^* mice resulted in a ~1.7-fold increase in atherosclerosis plaque area compared to the mice that received the control BM. However, the lesions in the mice that received *Abca1/g1* KO BM has significantly larger lesions (~3-fold) compared to control and *Mac^ABCDKO^* mice (~1.7-fold). This implies that there is a major effect of cholesterol efflux in cells upstream of macrophages in controlling atherosclerosis, likely HSPCs.

The studies of Westerterp et al., in the *Mac^ABCDKO^* mice also provided a novel insight on effects of altered cholesterol homeostasis in regulating the production of monocytes from the BM. Macrophages deficient in *Abca1/Abcg1* produced more M-CSF, G-CSF, and MCP-1, key cytokines involved in myelopoiesis and monocyte emigration from the BM (36). Interestingly, there was also a significant number of lipid-laden foam cells in the BM and spleen of the *Mac^ABCDKO^* mice, probably representing BM monocytes and macrophages. Consistent with this idea, there was a significant portion of the blood monocytes from the *Mac^ABCDKO^* mice that were loaded with cholesterol. Thus, deletion of *Abca1*/*Abcg1* in hematopoietic progenitors (i.e., GMPs) could predispose these and their daughter cells (monocytes) to accumulate cholesterol. This could result in monocytes carrying lipid into the atheroma and perhaps facilitating their maturation into M1 macrophages as the macrophages from the *Mac^ABCDKO^* mice also displayed enhanced inflammatory gene expression (Figure 1). Additionally, *in vitro* migration studies revealed a severe migratory defect of cultured macrophages deficient in *Abca1/Abcg1* (37), how this translates into the *in vivo* setting is unknown.

## Monocyte to Macrophage Differentiation

The origin of the macrophage itself is not always from a blood monocyte (38). Yolk-sac derived tissue macrophages can sustain their population under steady-state conditions, without recruitment of blood monocytes (39–41). After an inflammatory insult, blood monocytes can be recruited to increase the macrophage pool and to enhance the inflammatory response (39–41). In the heart, an organ with abundant resident macrophages that are established during embryonic development, it was found that CCR2^+^ Ly6-C^hi^ monocyte-derived macrophages coordinate the inflammatory response after cardiac injury by AngII infusion (39) or myocardial infarction (MI) (39). These Ly6-C^hi^ monocytes express *Nr4a1*, a transcription factor critical in the development of Ly6-C^lo^ monocytes (42), at low levels (43). However, in the healing phase after a MI, *Nr4a1* (Nur77) levels are increased permitting the maturation and differentiation of Ly6-C^hi^ monocytes into Ly6-C^lo^ monocyte/macrophages (43). These macrophages contribute to healing and tissue remodeling by producing factors such as TGF-β, IL-10, and VEGF-α. In essence, these studies revealed that the Ly6-C^hi^ monocyte orchestrate the initial inflammatory event, likely by forming M1 macrophages and then also develop into the reparative, M2-like macrophage (43).

Extending these key findings to the atherosclerotic lesion, as M1 macrophages can develop into M2 macrophages after *Nr4a1* induction (43), and deletion of *Nr4a1* results in M1 polarized macrophages and increased atherosclerosis (44), it is possible that the environment of the atherosclerotic lesion could affect the M1 macrophages resulting in a failure to upregulate *Nr4a1* and prevents the differentiation into M2 cells (Figure 1). It should also be noted that Ly6-C^lo^ monocytes do enter the lesion (45), and while these cells could become M2-like macrophages, they may not frequent the lesion in large enough numbers to make an impact.

Another newly discovered macrophage subset is the Mox macrophage. These macrophages are distinct to the classical M1 or M2 macrophage, as these cells display a unique gene expression profile with induction of redox-related genes including heme oxygenase-1 under the control of the transcription factor *Nrf2* (46). Mox macrophages also display a decrease in phagocytic and chemotactic capacity. Interestingly, both M1 and M2 macrophages can differentiate into the Mox macrophage when incubated with oxidized phospholipids. The *in vivo* relevance of these cells is noted as approximately 30% of all lesional macrophages are of the Mox phenotype.

## Hypercholesterolemia Influences HSPCs to Produce Atherogenic Macrophages

It is clear from animal studies that a hypercholesterolemic environment enhances the production of myeloid cells, namely monocytes, which contribute to atherogenesis. However, a hypercholesterolemic environment could also induce a “memory” effect in the HSPCs, which could also alter the function of their daughter cells. This hypothesis was recently explored by Seijkens and co-workers (47). Similar to our studies (4), they found that hypercholesterolemic *Ldlr^−/−^* mice had an expanded pool of HSPCs in the BM. Interestingly, when they harvested the BM from hypercholesterolemic *Ldlr^−/−^* mice and transplanted it competition with BM from normocholesterolemic mice, they found that the BM from the hypercholesterolemic mice had an enhanced propensity to produce myeloid cells (47). This was even observed in a normocholesterolemic environment. Evidence was provided to support the hypothesis that the hypercholesterolemic-primed HSPCs produced atherogenic (i.e., M1) macrophages as the macrophages from these HSPCs produced higher amounts of TNF-α, IL-6, and MCP-1. It was also found in the subsequent atherogenesis studies that hypercholesterolemic-primed HSPCs produced leukocytes that more readily entered the atherosclerotic lesion. This resulted in larger more macrophage-rich lesions.

The cBMT studies into hypercholesterolemic and normocholesterolemic mice suggest that there is a memory effect in the HSPCs (47). This idea was recently brought to light by Kampen et al., who discovered that BM harvested from WTD-fed mice has a loss of epigenetic control of key myeloid genes such as PU.1 and IRF8 (48). Transplantation of the BM from the WTD-fed mice into *Ldlr*^−/−^ recipients, like the studies of Seijkens et al., also resulted in larger lesion compared to recipient mice that received BM from chow fed donors. Consistent with the changes in PU.1 and IRF8, the WTD-conditioned BM produced more leukocytes, particularly of the myeloid variety. There were also signs of extramedullary hematopoiesis as the WTD-conditioned BMT mice had splenomegaly. However, one caveat of this study was the mice that received the WTD-conditioned BM-developed hyperglycemia, which has been shown to have independent effects on BM progenitors to induce monocyte production and contribute to atherosclerosis (49).

Another important point to note is that these studies either performed BMTs using total BM or the total pool of HSPCs and not just the long-term repopulating cells. Thus, as we have also noted, a predominant expansion of the multipotential progenitor 2 (MMP2) HSPCs that is thought to give rise to myeloid cells in *Apoe^−/−^* mice (4), it is possible that hypercholesterolemia-priming promotes the expansion of a subset of HSPCs that preferentially produces atherogenic myeloid cells.

## Early Myeloid Lineage Skewing in Atherosclerosis: Emerging concepts

The idea is emerging that signaling events in hematopoietic stem cells (HSCs) are able to influence lineage selection in these cells. Recently, it was discovered that HSCs express the M-CSF receptor and the engagement with M-CSF activated the myeloid master regulator, PU.1 (50). Injection of mice with LPS increased M-CSF levels and PU.1 expression in HSCs, which is likely to be the initiating step of myeloid lineage skewing in response to an infection. However, the LPS receptor TLR4 is also expressed on BM stem and progenitor cells (51) and could have been an additional contributor to the early lineage selection in these studies. Linking these findings with cholesterol metabolism, macrophages deficient in either *Abca1* and/or *Abcg1* express more TLR4 on their surface and like *Apoe^−/−^* macrophages are more responsive to TLR4 ligands (3, 52, 53). Thus, it is also conceivable that defective cholesterol efflux pathways in HSCs could lead to enhanced expression of TLR4 that could sense endogenous ligands, priming these cells to sense myeloid promoting cytokines. Whether ligands of pattern recognition receptors (PRRs) such as damage associated molecular pattern (DAMPs) (including S100A8/A9 and HMGB1), heat shock proteins, and modified LDL particles (54), some of which are increased in people with CVD, are present in the BM and bind to TLR4 on HSPCs is unknown. Assuming TLR4 ligands are present within the stem cell niche, it is possible that their interaction with TLR4 on HSPCs could downregulate *Abca1*, *Abcg1* (55), and *Apoe* (56) by the activation of IRF3, preventing LXR activating these target genes (57). This lead to increased cholesterol in the cell membrane and increased levels of cytokine receptors (4, 5). This could occur through the prevention of key feedback loops, such as activation of the E3-ubiquitin ligase c-CBL, which we recently reported was perturbed in progenitor cells lacking ABCG4 (32), and is also reported to downregulate the M-CSFR (58). Taken together, it is conceivable that defective cholesterol efflux and a hypercholesterolemic environment could influence the HSCs to respond to myeloid promoting cytokines to produce more monocytes that may have an altered function, which could ultimately contribute to the pool of inflammatory lesional macrophages in the atherosclerotic plaque.

Dissecting out the contribution of changes in the HSPCs to the function of the macrophage will be critical in further understanding the mechanisms contributing to not only atherogenesis but also lesion regression. The lesion milieu is also critically important, and is a dynamic environment with the newly recruited cells also contributing to and being influenced by the environment. However, taken together, the emerging theme from recent literature suggests that therapeutic interventions aimed at targeting HSPCs (i.e., cholesterol efflux pathways) may be an effective strategy to treat atherosclerosis by not only inhibiting monocyte production and entry into lesions but also to change the function/phenotype of the mature macrophage.

## Conflict of Interest Statement

The authors declare that the research was conducted in the absence of any commercial or financial relationships that could be construed as a potential conflict of interest.
